# Case Report: Overload of a cross-country skier in elite sports, somatoform autonomic disorder, and end-of-career support

**DOI:** 10.3389/fpsyg.2026.1767962

**Published:** 2026-04-15

**Authors:** Alexander Schorb, Carla Edwards, Wolfgang Aichhorn, Günter Schiepek

**Affiliations:** 1 University Hospital of Psychiatry, Psychotherapy and Psychosomatics, Paracelsus Medical University, Salzburg, Austria; 2 Department of Psychiatry and Behavioral Neurosciences, Michael G. DeGroote School of Medicine, McMaster University, Kitchener, ON, Canada; 3 Institute of Synergetics and Psychotherapy Research, Paracelsus Medical University, Salzburg, Austria

**Keywords:** International Society for Sports Psychiatry (ISSP), policy and advocacy for sports psychiatry and psychotherapy, sports psychiatry, sports psychotherapy, Synergetic Navigation System (SNS)

## Abstract

**Introduction:**

In recent years, sports psychiatry and psychotherapy has developed rapidly. Under the umbrella of the International Society for Sports Psychiatry (ISSP), the first international consensus statement on sports psychiatry was recently published. Fields of sports psychiatry were identified. In the field of competitive and elite sports, the end of a sports career is a vulnerable phase. The end of a sports career can lead to an increased prevalence of psychiatric symptoms and disorders. This is illustrated using the case of a cross-country skier.

**Case description:**

The interactions between success, mental health, stress, training and overload are presented. In this context, a treatment with the Synergetic Navigation System (SNS) for digitized real-time monitoring is presented, considering the interaction of the various factors. Symptoms of overtraining were apparently helpful in treating somatoform autonomic dysfunction. The pre-treatment proved helpful when the athlete returned for treatment years later due to depression at the end of his career.

**Discussion:**

To set a standard of service and a direction for future development in the field of sports psychiatry and psychotherapy, it is necessary to define typical and problematic issues to develop common approaches. The end of a career is an important issue in competitive and elite sports, and one in which athletes need support, recommended with integration into mental health care and prevention concepts.

**Conclusion:**

At the end of a sports career thorough and comprehensive preparation for post sport life is highly recommended. An “Exit Health Examination” including mental health exploration and “After Career Consultations” could be beneficial.

## Introduction

Sports psychiatry and sports psychotherapy have developed rapidly in recent years (Claussen et al., 2021). As part of a Summit of the International Society for Sports Psychiatry (ISSP), three fields of sports psychiatry were identified, namely: mental health and disorders in competitive and elite sports, mental health and sport-specific mental disorders in recreational sports, and sports and exercise in prevention of and treatment for mental disorders (Claussen et al., 2024). In the field of competitive and elite sports, the end of a sports career is a vulnerable phase. The end of a sports career can lead to an increased prevalence of psychiatric symptoms and disorders (Gouttebarge et al., 2019; Schorb and Schiepek, 2023). Many athletes, regardless of their background, may be affected (Claussen et al., 2021; Gouttebarge et al., 2019). Furthermore, athletes tend to seek treatment late and usually only when mental health issues become measurably impaired performance (Schorb et al., 2025). Prevention and care concepts are important and have been developed in the past (Schorb et al., 2025). Literature confirms that it is also beneficial for athletes to prepare for life after their career (Claussen et al., 2021; Gouttebarge et al., 2019; Schorb and Schiepek, 2023). Therefore, it would be useful to discuss appropriate care structures (Gouttebarge et al., 2019; Schorb and Schiepek, 2023; Schorb et al., 2025). The question arises as to whether clubs and federations should also get involved in providing support at the end of a career. The following case study of a cross-country skier illustrates a typical case in this context.

## Case of a cross-country skier in elite sports

A cross-country skier developed an infection and then a depressive episode immediately following a successful Olympic performance. The depression was not treated directly by a specialist, but by a practitioner in general practice. An arrhythmia developed, but no organic cardiological etiology was found. Detailed medical examinations were carried out, in particular to rule out any organic causes. These were performed accurately in somatic medicine and especially in cardiology, with no pathological findings. Since the condition significantly impeded training, the patient was referred to sports psychiatry and sports psychotherapy. Subsequently, a somatoform autonomic disorder was diagnosed, and treatment was started.

## Treatment of somatoform autonomic disorder with the synergetic navigation system (SNS)

The SNS was used to support treatment. This system is already used as a tool for mental health care and prevention in competitive sports (Schorb et al., 2025). The SNS is an Internet-based system used to record, visualize and analyze change processes and its non-linear trajectories (Schiepek and Strunk, 2010; Schiepek et al., 2019, 2022). It can be applied in diverse fields like psychotherapy, coaching, team development and in sports (Schiepek et al., 2023). The visualization and evaluation of the processes enable collaborative process feedback between therapists and athletes, as well as data-driven management of therapy, training and change processes. Questionnaires used for high-frequency self-assessments (e.g., day by day) provide data and time series analysis that can be used for reflection. In particular, they are used to visualize and conceptualize change processes in the data (Schiepek, 2022). The SNS can be used as a tool to guide effort, training and competition management to prevent non-functional overreaching and overtraining (Schorb and Schiepek, 2023; Schiepek et al., 2019; Schorb et al., 2021). Furthermore, it is suited to promote performance and to stabilize the psychological structure (Schorb et al., 2025; Schiepek et al., 2019, 2022; Schorb et al., 2021).

**Figure 1:**
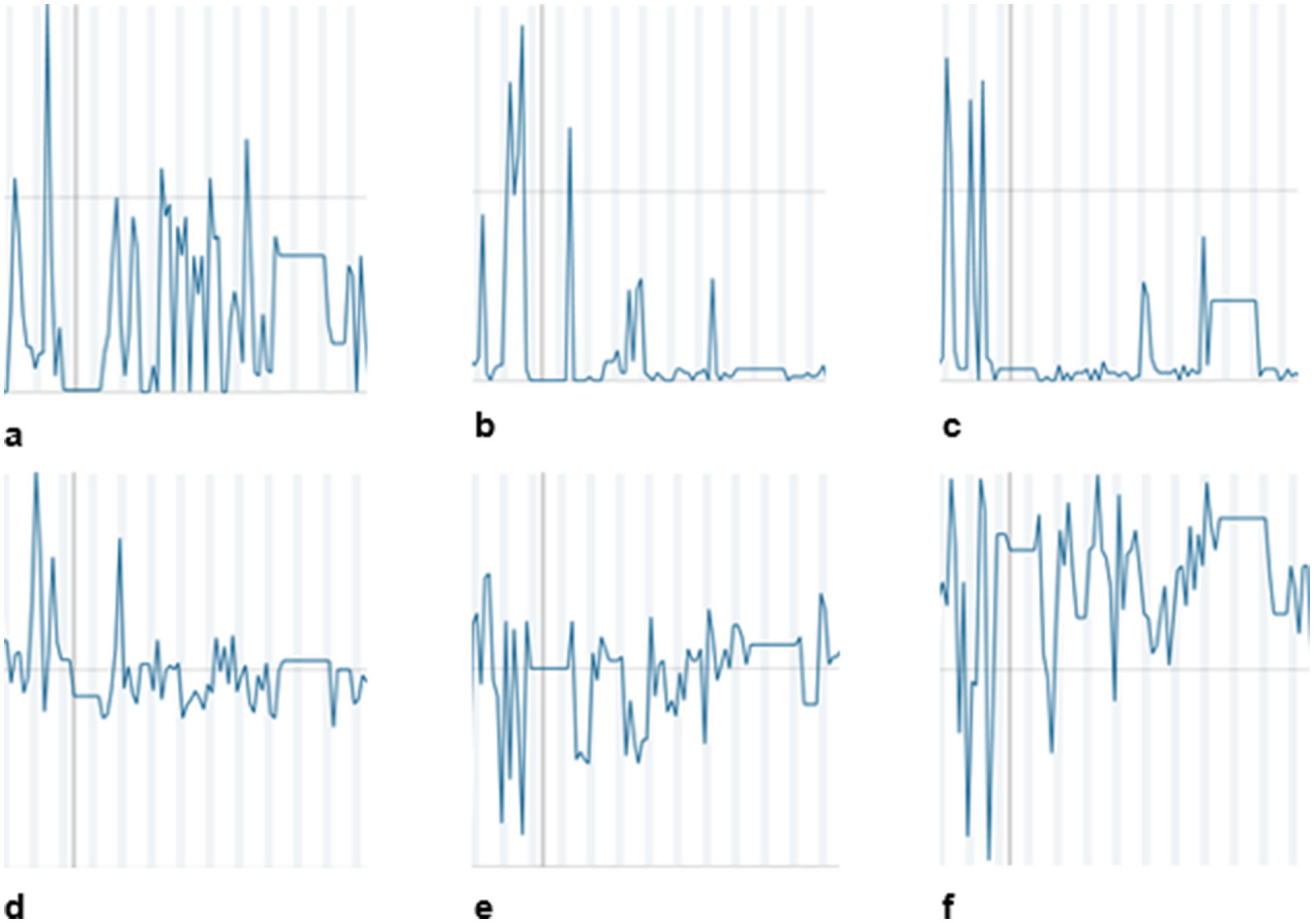
Process monitoring of a cross-country skier with the SNS. Time series of selected items of the “Therapy Process Questionnaire” (TPQ) (Schiepek et al., 2022) and “Sports Process Questionnaire” (SPQ) (Schiepek et al., 2023). X-axis: Time in units of days. Y-Axis: Intensity. **(a)** Training time per day. **(b)** Experienced sadness. **(c)** Experienced abnormalities in body function. **(d)** Factor Athlete Burnout (emotional and physical exhaustion / reduced athletic accomplishment / sport devaluation). **(e)** Self-esteem. **(f)** “Today I was satisfied with myself”.

Figure 1 shows a temporary overtraining state of the cross-country skier illustrated by the diagrams of the SNS (raw data time series) based on effort parameters and in the context of conspicuous mood values. During the preparation for the new season, the cross-country skier had an argument with his new coach regarding training load. An increase of load led to a temporal state of overtraining, which could be seen in the SNS.

**Figure 2:**
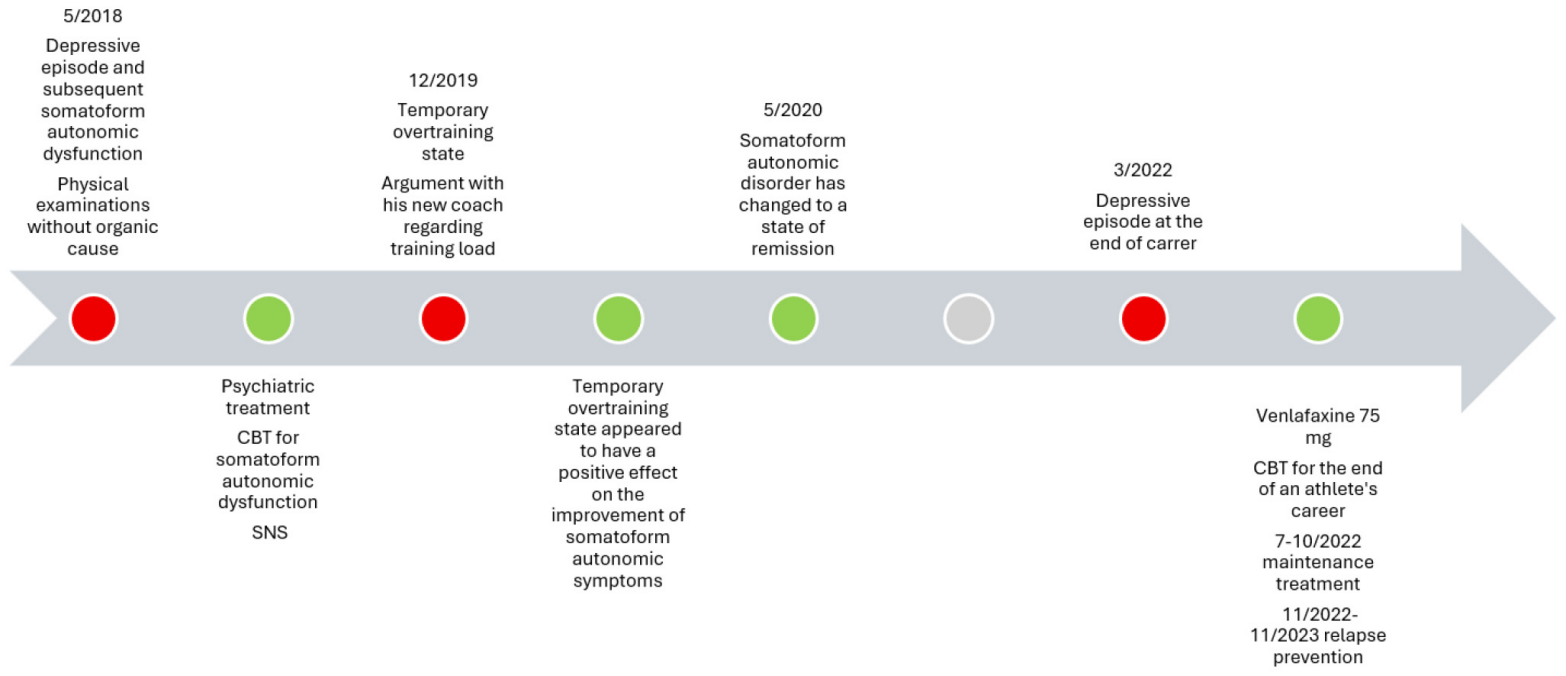
Clinical timeline of the presenting case.

As illustrated in Figure 1 graph (b), the athlete, who otherwise tended to overregulate his mood, showed an intensification of mood state in the SNS when he felt increasingly sad. This intensification occurred for a short period of time. After that, the intensity of the somatoform autonomic symptoms decreased, which was the reason for sports psychiatric and sports psychotherapeutic treatment. The main psychotherapeutic treatment method was cognitive behavioral therapy (CBT). CBT is an effective and evidence-based treatment for somatoform and autonomous functional symptoms. It treats these conditions by addressing the interplay between thoughts, emotions, and behaviors. CBT helps reduce anxiety and symptom severity by correcting misinterpretations of bodily sensations. In the case of the skier, the temporary state of overtraining with short-term negative affects and burnout symptoms facilitated a correct interpretation of the originally misinterpreted bodily sensations. However, the negative affective symptoms appeared only temporarily and did not require specific treatment. In the course of treatment and with the continuation of training and competitions the somatoform autonomous functional disorder successively changed to a remission state and the cardiac arrhythmia no longer occurred. The clinical timeline is shown in Figure 2.

Overall, the cross-country skier seemed much more confident about his sporting success in addition to demonstrating an increase in self-esteem. With this improvement in confidence, the athlete was able to reduce the frequency of therapeutic contacts and subsequently finish treatment.

## Treatment of depression at the end of career

Two years later, the patient returned due to a depressive episode. Contextual factors around this depressive episode included inability for the athlete to receive ongoing financial support and funding military service that would have been associated with his sport organization, since the position was intended for a junior athlete. In some sports, there are only a limited number of places for athletes. During this period of uncertainty, the patient experienced suicidal ideation that included a plan, however his family was identified as a strong protective factor. Acute psychiatric treatment was initiated, and functional support was provided by an external government agency that supports athletes. The patient underwent psychotherapeutic intervention (CBT) again as well as treatment with antidepressant medication (venlafaxine). CBT for the end of an athlete's career addresses the severe loss of identity, purpose, and social structure. It focuses on transitioning from a purely performance-driven mindset to a new life role by challenging cognitive distortions, managing anxiety, depression, and rebuilding self-worth outside of sports. Venlafaxine was administered as monotherapy. It was treated with a daily dose of 75 mg and was generally well tolerated. Maintenance treatment and relapse prevention involved combining long-term pharmacotherapy (full-dose antidepressants) with psychotherapeutic follow-up appointments to prevent symptom recurrence, identifying early warning signs of a new episode and a focus on stabilizing interpersonal relationships. The clinical timeline is shown in Figure 2.

The total duration of the treatment at the end of the career was 1 year and 5 months, including maintenance treatment and relapse prevention. For rational and practical reasons, the patient and therapist decided to treat without SNS at this stage, since the patient had already undergone this in a long process and the psychodynamics were well known. The treatment led to complete remission. After recovery, the athlete began studying sports and training sciences at university. He later became a coach himself and worked with young talents.

## Discussion

The case of the cross-country skier shows that this athlete, who obviously benefited in sports from overregulating his mood, benefited from an intensification of his mood due to an overtraining state in the case of somatoform autonomic dysregulation. The case shows also that support for athletes at the end of their careers can be necessary. Since the athlete had already been in contact with psychiatric and psychotherapeutic support structures long before the end of his career, he was able to receive rapid treatment during a subsequent depressive episode at the end of his career. Earlier contact with sports psychiatry and psychotherapy might facilitate help-seeking by athletes through challenges faced during the end-of-career phase. Therefore, mental health care and prevention projects can be helpful as part of a general, long-term care structure (Schorb et al., 2025).

A part of mental health care and prevention could be done with the SNS (Schorb et al., 2025). The SNS could also be used for load control (Schorb et al., 2021) and injury prediction (Schiepek et al., 2023). In this context, athletes would already be in regular contact with the sports psychiatrists and psychotherapists. If symptoms of a mental disorder occur at the end of a career, the “Therapy Process Questionnaire” (TPQ; Schiepek et al., 2019) can be added to the “Sports Process Questionnaire” (SPQ), which is used to predict or prevent injuries (Schiepek et al., 2023). To support change processes during psychotherapy, the TPQ, which had been validated in the past (Schiepek et al., 2019, 2022), is available to monitor psychotherapeutic processes at high-frequency sampling rates (daily self-assessments). The SNS is an open system in which, in principle, all conceivable questionnaires can be displayed at any frequency. For illustrative purposes, some items from the TPQ are listed in Table 1.

**Table 1:** Items from the “Therapy Process Questionnaire” (TPQ) (Schiepek et al., 2022) that could be included in the “Sports Process Questionnaire” (SPQ) (Schiepek et al., 2023) in the case of psychotherapeutic intervention.

Well-being and positive emotions (WPE)	
1	Today I felt joy (*not at all – very much*)
2	Today I experienced moments of happiness and light-heartedness *(not at all–very much)*
Emotional and problem intensity (EPI)	
1	Today I felt anxious (*not at all–very much*)
2	Today I felt tense and restless (*not at all–very much*)
3	Today I felt guilty (*not at all–very much*)
4	Today I felt shame (*not at all–very much*)
5	Today I felt angry (*not at all–very much*)
Insight/confidence/therapeutic progress (ICP)	
1	Today I became aware of relations that were not clear to me before (*not at all–very much*)
2	Today I gained insight into how my thoughts, feelings and behavior influence each other (*not at all–very much*)
3	Today I worked on things that were new and unusual for me (*not at all–very much*)

The scales used to answer the items are visual analog scales in which the scale range is translated into values from 0 to 100.

The functionality of retirement should be considered. The following three reasons are functional reasons for retirement: (a) tired of the circuit or lifestyle or time to move on, (b) achieved the goals, and (c) had difficulties with the coaching staff (Sinclair and Orlick, 1993). However, the end of a sports career often represents a significant stressor for athletes leading to an increased prevalence of psychiatric symptoms and disorders (Gouttebarge et al., 2019; Schorb and Schiepek, 2023). The enhanced access to support and services provided to athletes may potentially leave such individuals less vulnerable to feelings of isolation and helplessness once outside the athlete role (Jewett et al., 2018). Challenges experienced by athletes as they navigate their end-of-career phase include high athletic identity (Knights et al., 2016; Dimoula et al., 2013), feelings of loss and void (Stephan et al., 2003), finding a non-sport or sports related career plan, as well as adequate retirement planning (Perna and Ahlgren, 1999). Athletes with vocational plans have a higher degree of life satisfaction (Martin et al., 2014). The greatest risk of adjustment issues occurs with involuntary retirement (Martin et al., 2014) and unplanned retirement (Stambulova et al., 2007). When retirement is planned, athletes have a higher cognitive, emotional and behavioral readiness (Alfermann et al., 2004). Achieving sport-related goals tends to make retirement easier (Sinclair and Orlick, 1993). Measures to support retirement can be useful. Measures for social adjustment to the post-sport stage of life are listed in Table 2.

**Table 2:** Measures for social adjustment to the post-sport stage of life (Shutova et al., 2019; Carmody et al., 2019; Gouttebarge et al., 2018).

Measures for social adjustment to the post-sport stage of life	
1	Creation of conditions for future career guidance and training still during the active sports career as part of a mental health and prevention care structure
2	Exit Health Examination (EHE)
3	After Career Consultations (ACC)
4	Formation of a support system for employment of athletes who have completed their sports career
5	Variable models of psychological and pedagogical support for athletes completing a professional career
6	Athletes could contribute their experience and potential gained during their time as athletes in their new professions after their careers

The premise behind these approaches is to solve the negative effects of the transition of elite athletes to another type of work by utilizing the potential that the athletes have acquired during their time of intense competition (Shutova et al., 2019). Research has also yielded recommendations that former elite athletes should prepare for post sport life (Gouttebarge et al., 2019) and that an “Exit Health Examination” (EHE) could be beneficial (Carmody et al., 2019). Other studies recommend “After Career Consultations” (ACC) as an important concept for the mental health of retired athletes (Gouttebarge et al., 2018). In this context, it would make sense to involve clubs and federations in financing care structures for end of career support.

## Conclusion

Psychiatric psychotherapeutic support should be offered regularly to athletes during their careers. In the present case, the resolution of overregulated emotional states in an elite athlete due to overtraining appeared to contribute constructively to the treatment of somatoform autonomic symptoms. At the end of a career, the prevalence of mental health symptoms and disorders is increased among elite athletes. A standard of care that offers access to support and, if necessary, treatment at the end of a sports career is recommended. If athletes have already received psychiatric psychotherapeutic treatment during their career, it seems to be easier for them to receive treatment again. It is highly recommended that former elite athletes prepare thoroughly for post sport life. An “Exit Health Examination” (EHE) including mental health exploration and further career counseling for life after sports including “After Career Consultations” (ACC) could be beneficial.

## Limitations

The Limitations of this case report are: No validated scale use, lack of the patient's perspective, no structured interview for diagnosis.

## Data Availability

The original contributions presented in the study are included in the article/supplementary material, further inquiries can be directed to the corresponding author.

## References

[B1] Alfermann D. Stambulova N. Zemaityte A. (2004). Reactions to sport career termination: a cross-national comparison of German, Lithuanian, and Russian athletes. Psychol. Sport Exercise 5, 61–75. doi: 10.1016/S1469-0292(02)00050-X

[B2] Carmody S. Jones C. Malhotra A. . (2019). Put out to pasture: what is our duty of care to the retiring professional footballer? Promoting the concept of the ‘exit health examination' (EHE). Br. J. Sports Med. 24, 788–789. doi: 10.1136/bjsports-2017-098392 29574450

[B3] Claussen M. C. Currie A. Koh Boon Yau E. Nishida M. Martínez V. Burger J. . (2024). First international consensus statement on sports psychiatry. Scand. J. Med. Sci. Sports 34:e14627. doi: 10.1111/sms.14627 38610076 PMC12810445

[B4] Claussen M. C. Gonzalez Hofmann C. Schneeberger A. R. Seifritz E. Schorb A. Allroggen M. . (2021). Position paper: sports psychiatric care provision in competitive sports. Dtsch Z Sportmed. 72, 316–322. doi: 10.5960/dzsm.2021.503

[B5] Dimoula F. Torregrosa M. Psychountaki M. Gonzalez Fernandez M. D. (2013). Retiring from elite sports in Greece and Spain. Spanish J. Psychol. 16, 1–11. doi: 10.1017/sjp.2013.18 23866233

[B6] Gouttebarge V. Castaldelli-Maia J. M. Gorczynski P. Hainline B. Hitchcock M. E. Kerkhoffs G. M. . (2019). Occurrence of mental health symptoms and disorders in current and former elite athletes: a systematic review and meta-analysis. Br. J. Sports Med. 53, 700–706. doi: 10.1136/bjsports-2019-100671 31097451 PMC6579497

[B7] Gouttebarge V. Goedhart E. Kerkhoffs G. (2018). Empowering the health of retired professional footballers: the systematic development of an after career consultation and Its feasibility. BMJ Open Sport Exerc. Med. 4:e000466. doi: 10.1136/bmjsem-2018-000466 30774974 PMC6350730

[B8] Jewett R. Kerr G. Tamminen K. (2018). University sport retirement and athlete mental health: a narrative analysis. Qual. Res. Sport Exercise Health 11, 416–433. doi: 10.1080/2159676X.2018.1506497

[B9] Knights S. Sherry E. Ruddock-Hudson M. (2016). Investigating elite end-of-athletic-career transition: a systematic review. J. Appl. Sport Psychol. 28, 291–308. doi: 10.1080/10413200.2015.1128992

[B10] Martin L. Fogarty G. Albion M. (2014). Changes in athletic identity and life satisfaction of elite athletes as a function of retirement status. J. Appl. Sport Psychol. 26, 96–110. doi: 10.1080/10413200.2013.798371

[B11] Perna F. Ahlgren R. (1999). The influence of career planning, race, and athletic injury on life satisfaction among recently retired collegiate male athletes. Sport Psychol. 13, 144–156. doi: 10.1123/tsp.13.2.144

[B12] Schiepek G. (2022). Prozessfeedback und Prozesssteuerung in der Psychotherapie. Erfahrungen mit dem Synergetischen Navigationssystem (SNS). Psychopraxis.Neuropraxis 25, 323–331. doi: 10.1007/s00739-022-00843-3

[B13] Schiepek G. Schöller H. Aichhorn W. Kratzer L. Goditsch H. Viol K. . (2022). Prozessmonitoring in der Psychotherapie: Der Therapie-Prozessbogen und seine psychometrischen Eigenschaften. Familiendynamik 47, 210–224. doi: 10.21706/fd-47-3-210

[B14] Schiepek G. Schorb A. Schöller H. Aichhorn W. (2023). Prediction of sports injuries by psychological process monitoring. Sports Psychiatry 2, 1–8. doi: 10.1024/2674-0052/a000038

[B15] Schiepek G. Stöger-Schmidinger B. Kronberger H. Aichhorn W. Kratzer L. Heinz P. . (2019). The therapy process questionnaire. Factor analysis and psychometric properties of a multidimensional self-rating scale for high-frequency monitoring of psychotherapeutic processes. Clin. Psychol. Psychother. 26, 586–602. doi: 10.1002/cpp.2384 31153157 PMC6852168

[B16] Schiepek G. Strunk G. (2010). The identification of critical fluctuations and phase transitions in short term and coarse-grained time series—a method for the real-time monitoring of human change processes. Biol. Cybern. 102, 197–207. doi: 10.1007/s00422-009-0362-1 20084517

[B17] Schorb A. Häfelinger U. Pesicka M. Kohl S. Karus F. Kaiser A. . (2025). Promoting mental health care to reach optimal performance in sports. Sports Psychiatry 4, 1–6. doi: 10.1024/2674-0052/a000106

[B18] Schorb A. Niebauer J. Aichhorn W. Schiepek G. Scherr J. Claussen M. C. . (2021). Overtraining from a sports psychiatry perspective. Dtsch. Z. Sportmed. 72, 271–279. doi: 10.5960/dzsm.2021.496

[B19] Schorb A. Schiepek G. (2023). Psychische Gesundheit und Sport. Familiendynamik 48, 318–329. doi: 10.21706/fd-48-4-318

[B20] Shutova T. Stolyar K. Vysotskaya T. (2019). Socio-psychological problems of highly trained athletes upon completion of sports career. J. Phys. Educ. Sport 19, 652–657. doi: 10.7752/jpes.2019.01094

[B21] Sinclair D. Orlick T. (1993). Positive transition from high-performance sport. Sport Psychol. 7, 138–150. doi: 10.1123/tsp.7.2.138

[B22] Stambulova N. Stephan Y. Japhag U. (2007). Athletic retirement: a cross-national comparison of elite French and Swedish athletes. Psychol. Sport and Exercise 8, 101–118. doi: 10.1016/j.psychsport.2006.05.002

[B23] Stephan Y. Billard J. Ninot G. Delignieres D. (2003). Repercussions of transition out of elite sport on subjective well-being: a one-year study. J. Appl. Sport Psychol. 15, 345–371. doi: 10.1080/714044202

